# Effects of the prebiotic lactoferrin on multidrug-resistant *Escherichia coli* infections in broiler chickens

**DOI:** 10.14202/vetworld.2021.2197-2205

**Published:** 2021-08-25

**Authors:** Heba Badr, Nehal M. Nabil, Maram M. Tawakol

**Affiliations:** Department of Bacteriology, Reference Laboratory for Veterinary Quality Control on Poultry Production, Animal Health Research Institute, Agricultural Research Center, Nadi El-Seid Street, Dokki, Giza 12618, Egypt

**Keywords:** chicken, electron microscope, lactoferrin, multidrug resistance *Escherichia coli*, prebiotic

## Abstract

**Background and Aim:**

Increased multidrug resistance in *Escherichia coli* has created challenges for the poultry industry. Consequently, new antimicrobial agents should preferentially be utilized for the prevention and treatment of *E. coli* outbreaks. This study aimed to evaluate the effects of lactoferrin (LF) as a prebiotic on broiler chicks challenged with multidrug-resistant *E. coli* in comparison with antibiotics.

**Materials and Methods:**

A total of 70 diseased flocks from Egypt were collected for *E. coli* isolation and identification, serotyping, and antimicrobial susceptibility pattern determination. *E. coli* was isolated and characterized phenotypically and one isolate that showed multidrug-resistance was selected. A challenge trial was performed to evaluate the effectiveness of LF as a prebiotic on the isolated multidrug-resistant *E. coli*. Liver samples were collected from the experimental chicks and subjected to *E. coli* enumeration to illustrate the effectiveness of LF on the liver cells and bacteria using an electron microscope. Serum samples were also collected to estimate lysozyme and nitric oxide (NO) concentrations.

**Results:**

After isolation of *E. coli* with a percentage of 54.3% from the diseased broilers, the strain was serotyped (identified serotypes: O2, O18, O55, O78, O86a, O111, O125, O126, O127, O157, O159, and O166). Multi-antibiotic resistance was found to be harbored in a high percentage among 11 antibiotic discs. The LF in the prophylactic and treated groups was found to have a significant effect in comparison with the group treated with the drug of choice (ciprofloxacin). Furthermore, a significant difference in the NO (one of non-specific immune response) and a non-significant difference in lysozyme concentrations were reported in the group fed on rations with LF in comparison with the non-fed group.

**Conclusion:**

LF was thus identified as an effective prebiotic that can improve chick performance, help them to overcome multidrug-resistant *E. coli* and stimulate immunity.

## Introduction

Most *Escherichia coli* strains are commensals that can cause intestinal disease in poultry called colibacillosis [1], which results in significant losses for the poultry industry; about 30% every year [2]. Colibacillosis produces different pathologic syndromes in broilers, such as respiratory colibacillosis, acute colisepticemia, yolk sac infection, air sacculitis, perihepatitis, and pericarditis [3].

Various antibiotics are used to prevent and control microbes such as *E. coli*, but this has led to the increased spread of drug-resistant *E. coli* strains [4]. The emergence and rapid dissemination of antibiotic-resistant bacteria have reduced the effectiveness of antibiotics and furthermore, they are also transmitted to humans via food chain [5]. Subsequently, there has been research [6] to evaluate prebiotics which have a wide range of mechanisms for the elimination of pathogens such as *E. coli* in chickens and may also improve growth and performance.

The prebiotic lactoferrin (LF) is an iron-binding glycoprotein that is related to the transferrin family which plays an important role in antimicrobial activity functions due to its unique structure [7], and it possesses immunomodulatory activities [8]. Moreover, LF has previously been shown to improve the performance of poultry [9]. Improving beneficial bacterial populations can help to protect the host against a wide range of pathogenic bacteria and viruses [10]. LF has antimicrobial activity which reported due to iron deprivation through the removal of an essential substrate required for bacterial growth, another activity of LF was bacteriostatic effect [11] by degrading the peptidoglycans in the bacterial cell wall affecting the membrane permeability resulting in cell lysis [12].

LF is derived from a variety of sources, including secretory fluids such as exocrine glands (maternal milk or tears), mucous secretions, and secondary granules of neutrophils and blood [13], and it is regarded as a potential pre-immune host defense system [14]. Lysozyme is an enzyme involved in the immune system that can hydrolyze the cell wall peptidoglycans of some micro-organisms leading to lysis at the time of phagocytosis [15]. In addition, lysozyme can also regulate immune function by directly or indirectly modulating the complement system and it can enhance the function and proliferation of polymorphonuclear neutrophils and phagocytes [16]. Nitric oxide (NO) is synthesized in animals and it response to inflammatory or pro-inflammatory mediators. The expression of NO may be beneficial in host defense or in modulating the immune response [17].

The aim of this study was to investigate if LF as a prebiotic could effectively treat broiler chickens challenged with multidrug-resistant *E. coli*, and if it was a suitable replacement for the antibiotic of choice (ciprofloxacin).

## Materials and Methods

### Ethical approval

The infection and treatment of the broiler chickens in this study were in accordance with the regulations for the care and husbandry of experimental animals and approved by the Animal Care Committee of the Animal Health Research Institute (AHRI) - Dokki, Giza, Egypt.

### Study period and location

The study was conducted from January 2019 to November 2020 in Reference Laboratory for Veterinary Quality Control on Poultry Production, Animal Health Research Institute- Dokki, Giza, Egypt.

### Sample collection

Chickens from seventy diseased broiler farms (5 chickens/farm; the age of the birds varied from 7 to 35 days) that suffered from ruffled feathers, depression, and loss of appetite were collected in Dakahlia and Sharkia Governorate, Egypt. The birds were subjected to post mortem examinations under septic conditions and the lesions were categorized as being caused by colisepticemia, air sacculitis, perihepatitis, and pericarditis. Samples from the internal organs (liver, lung, spleen, and heart) of each bird were collected in accordance with the Reference Laboratory for Veterinary Quality Control on Poultry Production (RLQP), and then pooled together for bacterial examinations.

### *E. coli* isolation and identification

*E. coli* was isolated and identified using different media (MacConkey agar, Eosin Methylene agar, Simmon citrate, Triple sugar iron, indol test, and oxidase test) according to a method described by Lee and Nolan [18].

The serotyping of the isolated *E. coli* was conducted with Somatic (O) antigens of *E. coli* using an *E. coli* antiserum kit (Denka Seiken Co., Tokyo, Japan), according to the manufacturer’s instructions.

### Antimicrobial sensitivity test (AST)

An AST was conducted for all isolates using a disk-diffusion test, according to a method described by Koneman *et al*.[19], against 11 antibiotics (Oxoid®, England, UK), which were as follows: “Amoxicillin (AM 10 μg), ampicillin (AMP 10 μg), ciprofloxacin (CIP 5 μg), clindamycin (DA 2 μg), colistin sulfate (CT 10 μg), erythromycin (E 15 μg), florfenicol (FFC 30 μg), norfloxacin (NOR 10 μg), streptomycin (S 10 μg), sulfamethoxazole-trimethoprim (SXT 25 μg), and tetracycline (T 30 μg),” and interpreted according to CLSI/NCCLS [20].

### *In vivo* assay of LF effect in experimentally infected chicks

The experiment was designed to investigate the effects of LF in parrel with the ciprofloxacin on chicks infected with the multidrug-resistant *E. coli* isolate, and they were carried out for two weeks in September 2020. The experiments were performed using one-day old chicks cobb, each group in separate cage at AHRI, Dokki, Egypt, feed weighted daily and added *ad libitum*.

#### Preparation of E. coli inoculum and oral challenge

The selected *E. coli* strain was O127, as it showed multidrug resistance with sensitivity only to ciprofloxacin. *E.coli* was inoculated in buffered peptone water broth aerobically for 24 h at 37°C. The broth was diluted with sterile saline and adjusted using a spectrophotometer (OD.600 wave-length) to 10^8^ colony forming units (CFU/mL) according to a method described by Wang *et al*. [21]. Chicks were orally challenged with 1 mL of 10^8^ CFU/mL *E. coli* using a sterile syringe for 4 groups (Table-1).

**Table-1:** Experimental design of the challenged chicks.

Group No.	No. of chicks	*Escherichia coli* challenge (10^8^ colony forming units/mL)	Lactoferrin or antibiotic application
Group 1 Negative control	10	-	-
Group 2 Positive control	10	3^rd^ day	-
Group 3 Prophylactic treatment	10	7^th^ day	3^rd^ day (Lactoferrin)
Group 4 Antibiotic treatment	10	3^rd^ day	7^th^ day (Antibiotic)
Group 5 Lactoferrin treatment	10	3^rd^ day	7^th^ day (Lactoferrin)

#### Experimental chicks

Fifty-five commercial Cobb chicks (1 day old) were housed in isolation cabinets. The chicks under experiment (50 chicks) were divided into five equal groups. Five chicks were examined for absence of *E. coli* infection using their internal organs. Chicks were not fed for 12 h to reduce the crop bulk, thus challenge by flushing of *E.coli* in chicks of 3 days age at groups numbers 2, 4, and 5 but at 1 week age of group number 3 as shown in Table-1. Observations were made for 15 days and the birds were fed *ad libitum* starter feed with 24 h of light daily. The prebiotic LF, we used Pravotin manufactured by Hygint comp., Alexandria, Egypt, Batch No.20037. Pravotin consisted of LF 100 mg, dextrose anhydrous, sucralose, maize starch, Aerosil 200 (silicon dioxide), and magnesium stearate. It was added to the feed at a dose of 0.1 g/kg, in accordance with a previously documented method [10], in group No. 3 and 5 at 3 and 7 days of age, respectively. In addition, an antibiotic treatment using ciprofloxacin (10%) in group (4) with a dose of 10 mg/kg body weight; 1 ml/liter of drinking water for 5 successive days. Ciprofloxacin was selected according to the results of the AST for the selected multidrug-resistant *E. coli* strain, which showed additional resistance to more than 11 antibiotics (previously mentioned); the additional resistance antibiotics for this strain were cephalexin (CL30 μg), aztreonam (AT 30 μg), cephalothin (KF 30 μg), cefotaxime (CTX 30 μg), ceftazidime (CAZ 30 μg), amoxicillin/clavulanic acid (AMC 30 μg), ceftriaxone (CRO 30 μg), gentamycin (CN 10 μg), and nalidixic acid (NA 30 μg).

The chicks were observed once a day until the end of the experiment at 15 days of age, at which point all chicks were euthanized. The performance parameters such as body weight and feed conversion ratio were observed; Clinical signs were noticed daily, and any dead chicks were subjected to a PM examination with a record of the feed intake. Finally, samples from the chick’s liver were collected from each chick individually and subjected to an *E. coli* count as following: one gram of liver from each chick was ground in sterile saline and then put in 9 ml saline, ten-fold serial dilutions were made up to (10^5^). One ml of each dilution was plated on Eosin Methylene blue by using the spreading method and incubated at 37^o^C for 24h according to a method described by EL-Sawah *et al*. [22], and then the suspected colonies were counted and identified.

### Transmission electron microscope

The transmission electron microscopy (TEM) lab FA-CURP, Fac. of Agric., Cairo University Research Park “FARP” was utilized for this investigation. TEM was carried out to detect the effects of LF on the infected chick’s liver with *E. coli* as a prophylactic and the treatment was compared with the ciprofloxacin treatment according to a method described by Mikhail *et al*. [23] as follow;

Preparation of liver tissue for examination with electron microscope: Slice tissue samples into ~ 1 mm slices were transferred to a separate vial to be fixed in 2.5% Glutaraldehyde and 1% of osmium-tetroxide (OsO4), dehydrated in an ethanol series and embedded in an epoxy resin. Microtome sections preparation: Samples were then sectioned (500-1000 μm thick) with ultra-microtome (Leica Ultracut UCT, Japan), sections were stained with Toluidine blue (1X) then sections were examined by camera Leica (model ICC50 HD). Samples were then Ultra-thin sectioned (90 μm thick) with the ultra-microtome mounted on copper grids (400 mesh). Sections were stained with 5% uranyl acetate and lead citrate and then allowed to dry well. Then stained copper grids were examined by transmission electron microscope JEOL (JEM-1400 TEM) at the candidate magnification. Images were captured by CCD camera model AMT, optronics camera with 1632 x 1632 pixel format as side mount configuration. This camera uses a 1394 firewire board for acquisition.

### Measurement of the non-specific immune response parameters

To examine the immune responses in the collected serum samples, 20 chicks that were 7 days old were divided into two groups, ten chicks per group. The first group was supplemented with rations that were free from additives (group No. 1 of the experiment) and the other group was fed rations that contained LF (group No. 3 of the experiment before inoculated with *E. coli*) with a dose of 0.1 g/kg, as described by Mohamed and Younis [10].

The detection of the lysozyme concentrations in the blood serum was in accordance with a method described by Ramadan and Attia [24]; Lysozymes were diffused through a 1% agarose gel containing a suspension of *Micrococcus lysodeikticus*, a clear zone ring of the lysis developed in the initially translucent agarose gel (each filled plate contained the 5 working lysozyme standards as well as the samples to be assayed). the diameter of the clearance zone around each well was measured to the nearest 0.1 mm with an enlarger- viewer (Kalestted-Laboratories Inc., TX, USA). A standard curve was developed for lysozyme standard solution at concentration of 3, 15, 30, 60 and 120μg/ml. Log of concentration was plotted against diameter of corresponding cleared zone on a semi-logarithmic graph.

The detection of the NO concentrations in the blood serum was in accordance with a method described by Ramadan and Attia [24]; In an acid medium and in the presence of nitrite, the nitrous acid diazotize-sulfanilamide and the product from 100μl of serum sample were coupled with 100μl N- (1-naphthyl) naphthylamine. The resulting azo dye has bright reddish- purple color which can be measured at 570 nm using an ELIZA reader after the mixture was incubated at 51ºC for 10 min.

### Statistical analysis

Data were analyzed using IBM-Statistical Package for the Social Sciences statistics version 20, collected data are presented as the arithmetic mean and the data were expressed as mean±standard error and two different parameters were compared using independent sample T-tests and the effects were considered significant at p<0.05.

## Results

### *E. coli* isolation, identification, and serotyping

*E. coli* isolates were identified and analyzed from the internal organs (liver, lung, spleen, and heart) of diseased broiler chickens that were collected from 70 farms located in El-Dakahlia and El-Sharkia Governorates; 38 farms (54.3%) had positive *E. coli* isolations. The isolates were differentiated serologically and the different serotypes reported were as follows: (1) O2, (1) O18, (3) O55, (5) O78, (3) O86a, (3) O111, (9) O125, (2) O126, (8) O127, (1) O157, (1) 159, and (1) O166.

### Antimicrobial susceptibility patterns of the isolated *E. coli*

The results of the antimicrobial susceptibility testing showed that the majority of *E. coli* isolates were resistant to most of the antimicrobial agents used (Table-2). The levels of resistance varied, as amoxicillin, ampicillin, and clindamycin were 100%, erythromycin, streptomycin, and sulfamethoxazole-trimethoprim were 94.7%, florfenicol and tetracycline were 97.4% and 89.4%, respectively, while ciprofloxacin, colistin sulfate, and norfloxacin were 73.7%. In contrast, colistin sulfate, norfloxacin, ciprofloxacin, tetracycline, florfenicol, and sulfamethoxazole-trimethoprim showed low levels of sensitivity at 26.3, 18.4, 15.8, 5.3, 2.6, and 2.6%, respectively. The multidrug-resistant *E. coli* O127 strain selected for the experimental trials had to be sensitive to at least one antibiotic and easily applicable (per-os) and absorbed from the digestive system at young age.

**Table-2:** Antimicrobial Susceptibility pattern of the isolated *E. coli.*

Antimicrobial agent	*E. coli* (38 isolates)		

	Resistant No. (%)*	Intermediate No. (%)*	Sensitive No. (%)*
Amoxicillin (AM^10^)	38 (100)	0 (0)	0 (0)
Ampicillin (AMP^10^)	38 (100)	0 (0)	0 (0)
Ciprofloxacin (CIP^5^)	28 (73.7)	4 (10.5)	6 (15.8)
Clindamycin (DA^2^)	38 (100)	0 (0)	0 (0)
Colistin sulfate (CT^10^)	28 (73.7)	0 (0)	10 (26.3)
Erythromycin (E^15^)	36 (94.7)	2 (5.3)	0 (0)
Florfenicol (FFC^30^)	37 (97.4)	0 (0)	1 (2.6)
Norfloxacin (NOR^10^)	28 (73.7)	3 (7.9)	7 (18.4)
Streptomycin (S^10^)	36 (94.7)	2 (5.3)	0 (0)
Sulfamethoxazole-trimethoprim (SXT^25^)	36 (94.7)	1 (2.65)	1 (2.65)
Tetracycline (T^30^)	34 (89.4)	2 (5.3)	2 (5.3)

* Percentage calculated by dividing the result to total number of *E. coli* isolates. *E. coli*=*Escherichia coli*

### Experimental study

**Figure-1:**
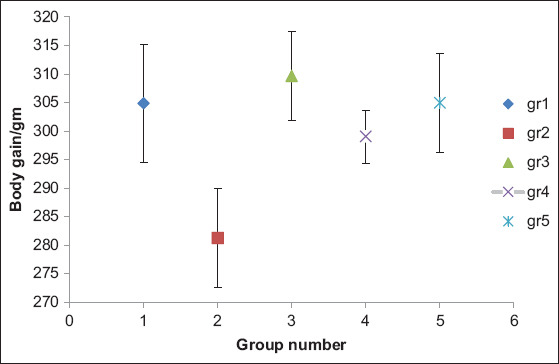
Body gain for chicks in all groups. Group 1: Negative control, Group 2: Positive control (infected with Escherichia coli), Group 3: Prophylactic treatment with lactoferrin, Group 4: Treated group with antibiotic and Group 5: Treated group with lactoferrin.

No mortalities were observed in the groups except for 20% of the positive control group (infected group) and 10% of the group treated with ciprofloxacin. Throughout the observation period, no clinical signs were seen with the negative control, prophylactic, or in the group treated with LF. The clinical signs were mild respiratory issues such as sneezing and coughing and brownish diarrhea that appeared 5 days post-challenge in the positive control group. In the PM examination, the 3 dead chicks (2 from positive control group and one from ciprofloxacin treated group) and euthanized positive control group showed enlarged intestine filled with diarrhea, turbid air sacs, and petechial hemorrhage at the liver surface, while the other groups observed with no P.M lesion on liver, intestine and airsac. There was a small significant variation between the weights of the chicks and the feed conversion rates after the experimental period. The prophylactic and LF treated groups (3 and 5, respectively) were nearly or slightly higher than the negative control group while a high performance was found in group No. 3 (prophylactic treatment with LF) throughout the rearing period (Table-3). Figure-1 presents the weight gain of each chick in each group.

**Table-3:** Body weight and Feed conversion rate of chicks at the end of experiment.

Group	Body gain (g) (mean±standard error)	Feed intake (g) (mean±standard error)	Feed conversion rate
Group 1			
Negative control	304.8±10.358	5300±8.87163	17.38845
Group 2			
Positive control (challenged with *E. coli)*	281.2±8.71525	4800±6.961	17.0697
Group 3			
Challenged group with Lactoferrin prophylactic treatment	309.6±7.80598	5500±6.27553	17.76486
Group 4			
Challenged group with ciprofloxacin treatment	299±4.6428	5200±5.40473	17.3913
Group 5			
Challenged group with Lactoferrin treatment	304.9±8.73626	5400±6.89194	17.71072

*E. coli=Escherichia coli*

### Enumeration of *E. coli* in the experimental group

The enumeration of the *E. coli* in the liver of each chick after it was euthanized and in the dead chicks showed: no *E. coli* in Group 1 (negative control; not inoculated with *E. coli*) or Groups 3 and 5 (infected group with prophylactic and treatment with LF, respectively). In contrast, in Group 4 (infected group with ciprofloxacin treatment) there was a low *E. coli* count on the dead chick and the two euthanized chicks, as shown in Table-4.

**Table-4:** Total *E. coli* count in liver of the experimental chicks.

Chicks No.	*E. coli* count (colony forming units/g)				

	Group 1	Group 2	Group 3	Group 4	Group 5
1	0	4.6×10^6^	0	2×10^3^	0
2	0	2.8×10^6^	0	0	0
3	0	3.4×10^6^	0	0	0
4	0	5×106	0	0	0
5	0	5.6×10^6^	0	0	0
6	0	4.2×10^6^	0	0	0
7	0	1.6×10^6^	0	0	0
8	0	9×10^5^	0	0	0
9	0	2.1×10^6^	0	7×10^1^	0
10	0	5×10^5^	0	3×10^1^	0

*E. coli=Escherichia coli*

### Transmission electron microscope

**Figure-2:**
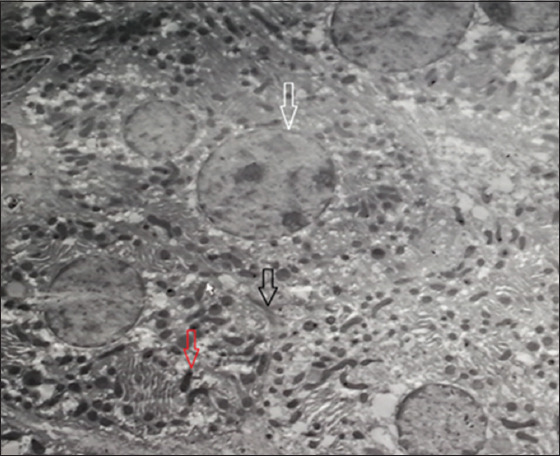
Group 1 (negative control) showed normal liver cells; black arrow shows the liver cell line, white arrow shows normal nucleus membrane and nucleus content and red arrow shows normal mitochondria with normal cytoplasm distribution.

**Figure-3:**
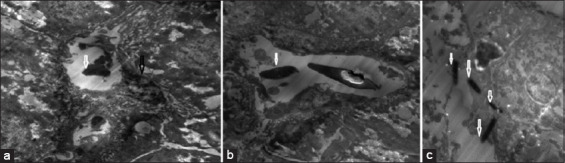
(a-c) Group 2 (infected group with *Escherichia coli*) all liver cell structures were destroyed; white arrows show the *E. coli* bacteria inside the liver cells and black arrow showed lysis nucleus.

**Figure-4:**
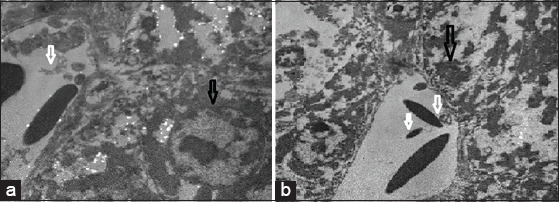
(a and b) Group 3 (infected group with *Escherichia coli* with prophylactic treatment with lactoferrin) black arrows showed repair action to the hepatic cells “nucleus and mitochondria” and white arrows showed lysis of bacteria cells.

**Figure-5:**
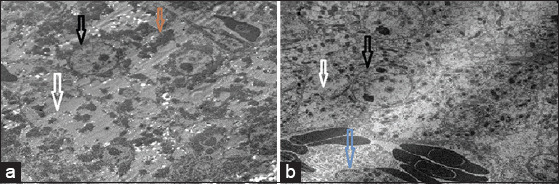
(a and b) Group 4 (infected with *Escherichia coli* and treated with ciprofloxacin) white arrow showed lysis of cytoplasm and cytoplasmic membrane with intact nucleus as shown in black arrow and damage of mitochondria moreover, appear the bacterial cell lysis as shown in brown arrow and blue arrow, respectively.

**Figure-6:**
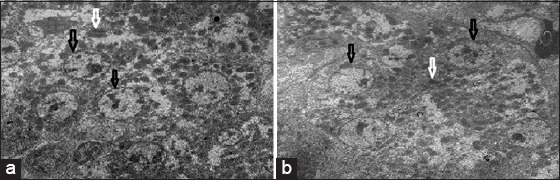
(a and b) Group 5 (infected with *Escherichia coli* with lactoferrin treatment) repair of cell line with intact nucleus and mitochondria as shown in black and white arrow, respectively.

Liver samples from the four groups were examined under an electron microscope and compared with the negative control (normal liver cells). It was found that the positive liver sample (infected liver with *E. coli*) showed an absence of liver cell structures, such as undistinguished membrane cell structures, damaged or shrunk nuclei, lysis of the cytoplasm, lysis of the mitochondria, and bacteria were found in the vacuolation and/or veins. In contrast, the liver sample treated by the LF in Groups 3 and 5 showed a better response than the ciprofloxacin treatment in Group 4. Groups 3 and 5 showed intact liver cell structures, with some damage of the mitochondria and lysis bacterial cells in the vacuole, while Group 4 showed lysis of the cytoplasm and noticeable improvement of the nucleus and mitochondria of liver cells (Figures-2-6).

### Measurement of the non-specific immune response parameters

Estimation of the lysozyme and NO concentrations in the blood serum indicates that the group supplemented with LF rations had increased lysozyme levels to variable degrees, using independent sample t-tests as a statistical method of calculation resulting in no significant difference of lysozyme concentration which is equal 0.16 (p>0.05) but the NO had slightly higher values in all of the examined serum samples when compared with the group that was feed rations without additives, which showed a highly significant difference (p<0.05). These results demonstrate that LF alone can have an effect on the immune response.

## Discussion

*E. coli* is a pathogen with a high incidence rate in intestinal and extra-intestinal disease cases in poultry [22]. APEC is considered a primary or secondary pathogen of poultry [25]. In the present study, 38 *E. coli* isolates were identified in the internal organs of 54.3% of the diseased broiler chickens assessed, which is similar to the isolation rates (53.4%, 52.26%, and 50.44% [25-27], respectively) from diseased broiler chickens that have previously been reported. However, some previous studies [28,29] have shown higher prevalence rates at 88.2% and 84%, respectively.

The serological identification conducted in this study reported 12 different serotypes (O2, O18, O55, O78, O86a, O111, O125, O126, O127, O157, O159, and O166). This was in accordance with a previous study [27] that also recorded 12 different serotypes, which were as follows: O1, O78, O126, O91, O125, O44, O121, O15, O146, O124, O20, and O128, and [30] in which the serological typing of 20 *E. coli* isolates revealed O27, O157, O26, O78, O6, O125, O44, O15, O115, O25, O168, O112a, and O63. In addition, another previous study [31] serotyped 40 strains that belonged to ten serovars, which were as follows: O78, O153, O168, O26, O157, O146, O20, O114, O125, and O126. These variable results indicate that there is a wide distribution of *E. coli* serotypes in diseased cases of broiler chickens and that most serotypes were incriminated in pathogenic disorders.

The misuse of antimicrobial agents has led to a rise of antimicrobial resistance against APEC, which is currently considered to be the main problem in the poultry industry [25]. In this study, the antimicrobial susceptibility testing reported that the majority of *E. coli* isolates were resistant to most of the antimicrobial agents currently used; 100% were resist to amoxicillin, ampicillin, and clindamycin followed by erythromycin, streptomycin, and sulfamethoxazole-trimethoprim were 94.7% resistance, florfenicol, and tetracycline which showed 97.4% and 89.4% resistance, respectively, while ciprofloxacin, colistin sulfate, and norfloxacin showed 73.7% resistance. A previous report [25] identified 95.9% resistance for florfenicol, 94.4% for amoxicillin, and 66% for ciprofloxacin. Furthermore, Enany *et al*. [27] found 100% resistance to ampicillin, erythromycin, and tetracycline, followed by norfloxacin (80.92%) and trimethoprim/sulfamethoxazole (75%), while 100% of the tested strains were sensitive to colistin sulfate. Oxytetracycline was recorded as having one of the highest levels of resistance, with a rate of 94.12%, followed by sulfamethoxazole + trimethoprim (88.89%), enrofloxacin (86.27%), and ampicillin (83.01%) but it differed in colistin sulfate, which showed lower resistance rates than our result of 6.54% [26].

The incidence of multidrug resistance among *E. coli* strains has hugely increased over the past two decades, and this has increased the search for novel antimicrobial strategies [32]. Several potential antimicrobial alternatives are currently being investigated, including probiotics, prebiotics, plant extracts, immunomodulatory, and antibacterial compounds [13]. LF is a natural cationic host defense protein that is utilized as a prebiotic and has been found to have several biological functions, including antimicrobial (inhibiting the growth of several of pathogenic bacteria, including “*E. coli* and antibiotic-resistant strains,” fungi, and even viruses), anti-cancer, antioxidant, and immunomodulatory effects in both *in vitro* and *in vivo* studies [33]. Studying the effects of LF on infected chicks with multidrug-resistant *E. coli* showed no mortalities in Groups 3 and 5 (infected group with prophylactic and treatment with LF) as well as the negative control group (uninfected group), in spite of the mortality in the positive control group (infected group) being 20% and that in the group treated with ciprofloxacin being 10%. Across the observation period, no clinical signs were seen in the negative control, prophylactic, and LF treated groups. However, mild respiratory signs, including sneezing and coughing and brownish diarrhea, appeared 5 days post-challenge in the positive control group. In the PM examination, the dead chicks (positive control group and ciprofloxacin treated group) and euthanized positive control group showed enlarged intestine filled with diarrhea, turbid air sacs and petechial hemorrhage at liver surface while the other groups were observed to be normal. A small significant variation was noticed between the groups for the weights of the chicks and the feed conversion rates after the experimental period; the prophylactic and treated groups with LF (Groups 3 and 5, respectively) are nearly or slightly higher than the negative control group while the high performance was found in group No 3 (prophylactic treatment with LF) during the rearing period. Other investigations have recorded similar results, as Yen *et al*. [33] performed pathogen challenges and one of the pathogenic bacteria tested was *E. coli*, which showed that the LF has broad-spectrum antimicrobial activity in the digestive tract and protects the mucosa of the small intestine from injury and results in significant improvement in weight gain; less severity of illness; lower bacterial load in the intestinal tract, blood and liver, and furthermore, this study suggested that LF combined with antibiotics is an important strategy for treating *E. coli* infections, especially those due to resistant strains. In addition, Edde *et al*. [34] pretreated neonate rats with hLF resulted in less bacteremia and lower disease severity scores than those not pretreated. In contrast, Geier *et al*. [13] investigated LF and the fact that it did not affect growth rate or feed conversion from 0 to 21 days of age, nor the performance or energy metabolism during the 7 days metabolism experiment which commenced at 25 days of age.

Examination of the liver sample under the electron microscope showed excessive damage; absence of the liver cell structure such as the undistinguished cell membrane structure, damaged or shrunk nuclei, lysis of the cytoplasm, lysis of the mitochondria, and the bacteria found in the vacuolation or veins in the infected liver with the *E. coli* in group (2). In contrast, the liver samples treated by LF in groups (3) and (5) showed the best response, with intact liver cell structures, and only some damage to the mitochondria and lysis of the bacterial cell in the vacuole, while the ciprofloxacin treatment in group (4) resulted in lysis of the cytoplasm and noticeable improvement of the nucleus and mitochondria of liver cells. Yen *et al*. [33] illustrated that the *E. coli* cells under the scanning electron microscopy, showed aggregative fragmentation, and displayed puncture holes with membrane breakdown after LF treatment for 2 and 4 h, respectively. The control groups of the *E. coli* without the LF treatment showed normal morphologies after 2 and 4 h incubations in the same conditions.

LF is involved in several physiological and protective functions, such as the regulation of iron absorption in the gut and has antioxidant, anti-cancer, and anti-inflammatory activities, as well as antimicrobial activities, which are its most studied function[11]. LF was identified in previous study as a feedstuff additive that enhances avian immunity[33] which illustrates the natural immune defense mechanisms, and perhaps that secretory IgA and lysozyme can overcome a pathogen invasion from the gastrointestinal tract [35]. In our study, estimations of the lysozyme and NO concentrations in the blood serum indicated that the group was supplemented with LF in rations and showed increases in the lysozyme of variable degrees, resulting in no significant difference which equal 0.16 (p>0.05) but the NO had slightly higher values in all the examined serum samples than another group were fed rations without additives, and showed highly significant differences (p<0.05). This data recognizes that LF alone can affect the immune response. Proof of this was previously presented [36] and these results clarified that LF serves as an immune mediator that naturally activates both the innate and adaptive immune functions. It is one of the first factors released by neutrophils by adjusting the target cell response, including those implicated in oxidative stress and systemic inflammatory responses. In addition, Stuehr and Ghosh [17] demonstrated that NO can also interact with reactive oxygen species, which mediates oxidative stress and is considered to be a pathway for biological effects, such as cytotoxicity.

These findings are supported by Cai *et al*. [7] who clarified the importance of LF in breast milk for the development of immunity and growth in infants. Whereas Edde *et al*. [34] suggested that LF may act with other natural peptides, such as lysozyme, or may prime macrophages to kill *E. coli* *in vivo*.

## Conclusion

The incidence of *E. coli* in poultry has increased with the aggressive increase in multidrug-resistant strains of *E. coli*. In addition to being a foodstuff, LF can cause significant improvements in weight gain, feed conversion rates, and the performance of chicks, as well as having antimicrobial effects for multidrug resistance strains of *E. coli*. This elaborate that a non-specific immune response is stimulated to be alert for any predisposing factors and suggests further study for immune response stimulated by LF.

## Authors’ Contributions

HB and NMN: Designed the study. HB, NMN, and MMT: Performed the research and drafted the manuscript. HB: Analyzed the data. HB, NMN, and MMT: Revised and finalized the manuscript for submission. All authors read and approved the final manuscript.

## References

[1] Bonnet C, Diarrassouba F, Brousseau R, Masson L, Topp E, Diarra M.S (2009). Pathotype and antibiotic resistance gene distributions of *Escherichia coli* isolates from broiler chickens raised on antimicrobial-supplemented diets. Appl. Environ. Microbiol..

[2] Barnes J, Gross B, Calnek W, Barnes J, Beard W, McDougald M, Saiﬁn M (1999). Colibacillosis. Diseases of Poultry.

[3] Catherine S, Schaeffer B, Brée A, Mora A, Dahbi G, Biet F, Oswald E, Mainil J, Blanco J, Moulin-Schouleur M (2012). Diagnostic strategy for identifying avian pathogenic *E. coli* based on four patterns of virulence genes. J. Clin. Microbiol.

[4] Elbaz A.M, El-Sheikh S.E (2020). Effect of dietary probiotic, antibiotic or combination on broiler performance, cecum microbial population and ileal development. Mansoura Vet. Med. J..

[5] Saleh A.A, Amber K, Mohammed A.A (2020). Dietary supplementation with avilamycin and *Lactobacillus acidophilus* effects growth performance and the expression of growth-related genes in broilers. Anim. Prod. Sci.

[6] Adhikari P.A, Kim W.K (2017). Overview of prebiotics and probiotics:Focus on performance, gut health and immunity a review. Ann. Anim. Sci..

[7] Cai X, Duan Y, Li Y, Wang J, Mao Y, Yang Z, Zhao X, Zhao Y, Guan Y, Yin S (2018). Lactoferrin level in breast milk:A study of 248 samples from eight regions in China. Food Funct.

[8] Vega-Bautista A, de la Garza M, Carrero J.C, Campos-Rodríguez R, Godínez-Victoria M, Drago-Serrano M.E (2019). The impact of lactoferrin on the growth of intestinal inhabitant bacteria. Int. J. Mol. Sci..

[9] Humphrey B.D, Huang N, Klasing K.C (2002). Rice expressing lactoferrin and lysozyme has antibiotic-like properties when fed to chicks. Nutr. J.

[10] Mohamed H.M.A, Younis W (2018). Trials on the role of prebiotics and probiotics in colonization and immune response of broiler chickens challenged with *Escherichia coli* K88. AJVS.

[11] Giansanti F, Panella G, Leboffe L, Antonini G (2016). Lactoferrin from milk:Nutraceutical and pharmacological properties. Pharm. J.

[12] Sahin O, Ziaei A, Karaismailoğlu E, Taheri N (2016). The serum angiotensin converting enzyme and lysozyme levels in patients with ocular involvement of autoimmune and infectious diseases. BMC Ophthalmol. J.

[13] Geier M.S, Torok V.A, Guo P, Allison G.E, Boulianne M, Janardhana V, Bean A.G.D, Hughes R.J (2011). The effects of lactoferrin on the intestinal environment of broiler chickens. Br. Poult. Sci.

[14] Kell D.B, Heyden E.L, Pretorius E (2020). The biology of lactoferrin, an iron-binding protein that can help defend against viruses and bacteria. Front. Immunol.

[15] Sahoo N.R, Kumar P, Bhusan B, Bhattacharya T.K, Dayal S, Sahoo M (2012). Lysozyme in livestock:A guide to selection for disease resistance:A review. J. Anim. Sci. Adv.

[16] Huang G, Li X, Lu D, Liu S, Suo X, Li Q, Li N (2018). Lysozyme improves gut performance and protects against enterotoxigenic *Escherichia coli* infection in neonatal piglets. Vet. Res.

[17] Stuehr D, Ghosh S, Bom G.Y.R, Cuatrecasas P, Ganten D, Herken H, Starke K, Taylor P (2000). Enzymology of nitric oxide synthases. Handbook of Experimental Pharmacology.

[18] Lee M.D, Nolan K.L, Zavala L.D, Swayne D.E, Glisson J.R, Jackwood M.W, Pearson J.E, Reed W.M (2008). A laboratory manual for the isolation and identification of avian pathogen. Editorial Board for the American Association of Avian Pathologists.

[19] Koneman E.W, Allen S.D, Janda W.M, Schreckenberger P.C, Winn W.C (1997). Diagnostic Microbiology.

[20] Clinical and Laboratory Standards Institute (2017). Performance Standards for Antimicrobial Susceptibility Testing.

[21] Wang S, Peng Q, Jia H.M, Zeng X.F, Zhu J.L, Hou C.L, Liu X.T, Yang F.J, Qiao S.Y (2017). Prevention of *Escherichia coli* infection in broiler chickens with *Lactobacillus plantarum* B1. Poult. Sci.

[22] El-Sawah A.A, Dahshan A.M, El-Nahass E, Abd El-Mawgoud A.I (2018). Pathogenicity of *Escherichia coli* O157 in commercial broiler chickens. Beni Suef Univ. J. Basic. Appl. Sci.

[23] Mikhail M.S, El-Banna O.M, Khalifa E.A, Mohammed A.M.S (2012). Detection and control of rose phytoplasma phyllody disease Egypt. J. Phytopathol.

[24] Ramadan A.A, Attia E.R.H (2003). Natural Killing Molecules in Cervical Mucus of Buffaloes during the Estrous Cycle.

[25] Ibrahim R.A, Cryer T.L, Lafi S.Q, Abu Basha E, Good L, Tarazi Y.H (2019). Identification of *Escherichia coli* from broiler chickens in Jordan, their antimicrobial resistance, gene characterization and the associated risk factors. BMC Vet. Res.

[26] Halfaoui Z, Menoueri N.M, Bendali L.M (2017). Serogrouping and antibiotic resistance of *Escherichia coli* isolated from broiler chicken with colibacillosis in center of Algeria. Vet. World.

[27] Enany M.E, Algammal A.M, Nasef S.A, Abo-Eillil S.A.M, Bin-Jumah M, Taha A.E, Allam A.A (2019). The occurrence of the multidrug resistance (MDR) and the prevalence of virulence genes and QACs resistance genes in *E. coli* isolated from environmental and avian sources. AMB Expr.

[28] El-Sukhon S.N, Musa A, Al-Attar M (2002). Studies on the bacterial etiology of air saculitis of broilers in Northern and Middle Jordan with special reference to *Escherichia coli*, *Ornithobacterium rhinotracheale,* and *Bordetella avium*. Avian Dis.

[29] Oboegbulem S, Abiade C, Onunkwo J, Ezenduka E, Chah F, Nwanta J, Anosike C (2009). Incidence of verotoxigenic *Escherichia coli* in poultry in Nsukka urban area of Southeastern Nigeria. Anim. Sci. Report.

[30] EL-Sawah A.A, Dahshan A.M, Nasef S.A, El-Nahass E, Nayel A.I (2016). Characterization of *E. coli* and *Salmonella* spp. isolates associated with omphalitis in baby chicks. J. Vet. Med. Res.

[31] Rezk M.M, Enany M.E, Hanafy M.S (2010). Relationship between O serogroup, virulence and plasmid profile in *Escherichia coli* isolated from diseased chickens. J. Food Saf.

[32] Nickel J.C (2007). Urinary tract infections and resistant bacteria:Highlights of a symposium at the combined meeting of the 25^th^ international congress of chemotherapy (ICC) and the 17^th^ European congress of clinical microbiology and infectious diseases (ECCMID), March 31-April 3, Munich, Germany. Rev. Urol.

[33] Yen C, Shen C, Hsu W, Chang Y, Lin H, Chen H, Chen C (2011). Lactoferrin:An iron-binding antimicrobial protein against *Escherichia coli* infection. Biometals.

[34] Edde L, Hipolito R.B, Hwang F.F, Headon D.R, Shalwitz R.A, Sherman M.P (2011). Lactoferrin protects neonatal rats from gut-related systemic infection. Am. J. Physiol. Gastrointest. Liver Physiol.

[35] Yen C.C, Lin C.Y, Chong K.Y, Tsai T.C, Shen C.J, Lin M.F, Su C.Y, Chen H.L, Chen C.M (2009). Lactoferrin as a natural regimen for selective decontamination of the digestive tract:Recombinant porcine lactoferrin expressed in the milk of transgenic mice protects neonates from pathogenic challenge in the gastrointestinal tract. J. Infect. Dis.

[36] Actor J.K, Hwang S, Kruzel M.L (2010). Lactoferrin as a natural immune modulator. Curr. Pharm. Des.

